# Most Effective Combination of Nutraceuticals for Improved Memory and Cognitive Performance in the House Cricket, *Acheta domesticus*

**DOI:** 10.3390/nu13020362

**Published:** 2021-01-25

**Authors:** Samskruthi Madireddy, Sahithi Madireddy

**Affiliations:** 1 Independent Researcher, 1353 Tanaka Drive, San Jose, CA 95131, USA; 2 Massachusetts Institute of Technology, Cambridge, MA 02139, USA; sahithim@mit.edu

**Keywords:** multivitamins, zinc, polyphenols, omega fatty acids, probiotics, improved memory, cognitive performance

## Abstract

Background: Dietary intake of multivitamins, zinc, polyphenols, omega fatty acids, and probiotics have all shown benefits in learning, spatial memory, and cognitive function. It is important to determine the most effective combination of antioxidants and/or probiotics because regular ingestion of all nutraceuticals may not be practical. This study examined various combinations of nutrients to determine which may best enhance spatial memory and cognitive performance in the house cricket (*Acheta domesticus* (L.)). Methods: Based on the 31 possible combinations of multivitamins, zinc, polyphenols, omega-3 polyunsaturated fatty acids (PUFAs), and probiotics, 128 house crickets were divided into one control group and 31 experimental groups with four house crickets in each group. Over eight weeks, crickets were fed their respective nutrients, and an Alternation Test and Recognition Memory Test were conducted every week using a Y-maze to test spatial working memory. Results: The highest-scoring diets shared by both tests were the combination of multivitamins, zinc, and omega-3 fatty acids (VitZncPuf; Alternation: slope = 0.07226, Recognition Memory: slope = 0.07001), the combination of probiotics, polyphenols, multivitamins, zinc, and omega-3 PUFAs (ProPolVitZncPuf; Alternation: slope = 0.07182, Recognition Memory: slope = 0.07001), the combination of probiotics, multivitamins, zinc, and omega-3 PUFAs (ProVitZncPuf; Alternation: slope = 0.06999, Recognition Memory: slope = 0.07001), and the combination of polyphenols, multivitamins, zinc, and omega-3 PUFAs (PolVitZncPuf; Alternation: slope = 0.06873, Recognition Memory: slope = 0.06956). Conclusion: All of the nutrient combinations demonstrated a benefit over the control diet, but the most significant improvement compared to the control was found in the VitZncPuf, ProVitZncPuf, PolVitZncPuf, and ProPolVitZncPuf. Since this study found no significant difference between the performance and improvement of subjects within these four groups, the combination of multivitamins, zinc, and omega-3 fatty acids (VitZncPuf) was concluded to be the most effective option for improving memory and cognitive performance.

## 1. Introduction

The healthcare community increasingly acknowledges the role of social determinants of health in driving health disparities. Food insecurity is one such factor that contributes to nutritional deficits, affecting both physical and mental health [1,2]. In 2016, about 11.5% of adults and 17.5% of children in the United States lived in households facing food insecurity [3]. This food insecurity carries a risk of many health issues common in the United States [4]. Access to nutritious food is important because evidence suggests that diet can play a significant role in cognition via the gut–brain axis, which is the bidirectional communication between the gut and the brain [5]. Moreover, certain foods are known to contain nutrients that can slow down cognitive decline or improve cognitive performance.

Individually, multivitamins, zinc, polyphenols, omega-3 fatty acids, and probiotics are shown to support brain function either through the gut–brain axis or through antioxidant functions [6,7,8,9,10]. However, the effect of various combinations of these nutrients on cognition and spatial memory remains unexplored. Knowing the most effective combination of antioxidants and/or probiotics is important, as regular ingestion of all nutraceuticals may not be practical. Finding the combination of nutrients that best enhances cognitive performance is crucial to developing strategies for improved learning and cognition in humans. This study examined the combinations of multivitamins, zinc, polyphenols, omega-3 fatty acids, and probiotics that best enhanced memory and cognitive performance in the house cricket.

## 2. Effect of Nutraceuticals

### 2.1. Polyphenols

Polyphenol intake has consistently shown benefits in various aspects of memory and learning [6,11]. Dietary polyphenols have been linked to greater cognitive evolution, as well as improvements in language and verbal memory [12,13]. These polyphenols are generally secondary plant metabolites with antioxidative properties. The antioxidant effects of polyphenols are important because the imbalance between antioxidants and reactive oxygen species (ROS) leads to oxidative stress [14,15,16]. The brain is particularly vulnerable to oxidative stress because of its high metabolic demand [17,18]. Because ROS are highly reactive, they frequently damage macromolecules, which can lead to mitochondrial dysfunction and ultimately neurodegeneration [19,20]. However, polyphenols have well-documented antioxidant and anti-inflammatory effects [21,22,23]. Polyphenols can protect against oxidative damage by scavenging free radicals and deactivating metals used in ROS generation [24,25,26]. They can also activate antioxidant enzymes, decrease peroxide levels, and repair membranes that have suffered oxidative damage [27].

### 2.2. Probiotics

In addition to polyphenols, probiotics can also improve cognitive function and spatial memory [9,28]. Probiotics refer to bacteria that support health by changing the composition of the gut microbiome [29]. This, in turn, influences brain health via the gut–brain axis [5,30]. Probiotics and gut microbes can affect brain physiology through their influence on cytokine levels [31]. Changes in the gut microbiota are communicated to the brain through the vagus nerve and through levels of dietary tryptophan, a serotonin precursor [32,33]. Dietary changes are one established method of altering gut microbiota populations [34]. Probiotics are capable of regulating the hypothalamic–pituitary–adrenal (HPA) axis [35], which is involved in the stress response, and changing levels of brain-derived neurotrophic factor (BDNF), which plays an important role in learning and memory [36].

### 2.3. Multivitamins

Similarly, multivitamins, especially vitamins A, C, and E, have been shown to benefit cognition and spatial memory as well [37,38,39]. Vitamins have been linked to better cognitive health, particularly in free recall memory [40,41]. Vitamins A, C, B group, and E can act as antioxidants by scavenging free radicals and preventing oxidative stress [42,43]. Vitamin C, in particular, is highly concentrated in the brain, where it promotes neuronal, vascular, and neurotransmitter function [44]. Decreased vitamin B12, B6, and folate have also been linked to cognitive decline associated with aging [45,46,47]. Moreover, maternal B12 levels have been linked to the cognitive function of their offspring [48]. Taking B vitamins was shown to be beneficial for the cognitive function of people without dementia as well [49]. Vitamin D is a steroid hormone with many functions that take effect after binding a receptor in the nucleus [50,51]. Vitamin D levels have also been linked to brain functioning [52]. This may be due to its role in neuroinflammation, which is involved in cognitive decline and neurodegeneration accompanying aging [53].

### 2.4. Omega-3 Polyunsaturated Fatty Acids

In addition, omega-3 PUFAs are important in neural function and they play a critical role as both energy substrates and cell membrane components [8,54,55]. They also protect against oxidative stress, inflammation, and apoptosis while mitigating the activity of neurotrophic factors [8]. Omega-3 PUFAs are essential in cellular function, as well as the development of cognition, learning, and memory [56,57]. Likewise, omega-3 fatty acids have been associated with increased relational memory, which is dependent on hippocampal brain activity [58]. Omega-3 fatty acids, including docosahexaenoic acid (DHA), have also been shown to improve synaptic plasticity, membrane fluidity, and neuronal metabolism [59,60,61,62]. PUFAs are involved in regulating glucose levels, feeding, neurotransmission, emotions, apoptosis, and neuroinflammation [63,64]. They also assist in behavior and cognitive development [65]. Fatty acids play an additional role in protecting against neuroinflammation and neuron death [66]. Therefore, neuronal function and integrity depend on adequate omega-3 PUFA levels. DHA, in particular, has been implied to play a role in preserving the health of aging neurons [67]. These effects may be through DHA’s ability to change the expression of genes that regulate neurogenesis and neuron function.

### 2.5. Zinc

Another nutrient, zinc, is crucial for memory formation and learning [68]. Zinc is necessary in forming synapses and in mediating structural plasticity; this activity potentially allows zinc to modulate the function of the hippocampus in memory [69]. Zinc is critical for cognitive development, since it is involved in neuronal migration and it regulates neurogenesis and differentiation [70,71]. In addition to its functions within glia and neurons, zinc also affects neurotransmission. Zinc levels in the brain are largely protected from zinc deficiencies in the diet since homeostasis is maintained by the blood–brain barrier and the blood–cerebrospinal fluid barrier [72]. Zinc deficiencies can affect attention, behavior, and motor development [73,74]. Animal studies have shown that psychological stress reduces serum zinc levels, implying that zinc deficiencies and gut inflammation are linked to stress [75]. Zinc is also essential in the general development and function of the central nervous system (CNS) [68]. Long-term administration of zinc sulfate in rats enhanced learning, spatial memory, and exploratory activity [10]. In addition to improving spatial working memory, zinc supplements in rats were also found to improve recognition memory [76]. Zinc might also affect memory formation through its ability to regulate glutamate signaling [76]. Additionally, zinc ions are highly concentrated in the hippocampus and, thus, play a key role in modulating spatial learning and memory [77,78].

## 3. Materials and Methods

### 3.1. Animals

Insects make suitable model organisms because they are less expensive, easier to maintain in bulk, and possess simpler nervous systems than vertebrates; however, they still share fundamental neurobiology and behavior with vertebrates [79]. Studies in insects have previously yielded significant insights about the fundamental processes behind learning and memory [80]. Moreover, studies using crickets have shown that mushroom bodies responsible for memory and olfactory learning in their brains continue neurogenesis into adulthood in response to sensory input [81,82,83]. This adult neurogenesis may be related to mechanisms of learning and memory in invertebrates and possibly vertebrates [84]. Crickets are useful as a model organism because they can retain an olfactory memory throughout their entire lives and modify it readily in response to experience [85]. Crickets are also a good model for nutritional effects. For example, one study used crickets to identify how protein and carbohydrate levels influenced weight, muscle mass, and fat reserves [86]. Crickets have been widely used within behavioral tests [87,88,89]. For example, one study used crickets with a plus-shaped maze to examine predator-induced stress and found that the crickets showed consistent behavioral responses in their tests [90]. Another study used the Y-maze with crickets to assess the role of thorax temperature during mate choice [91].

**Figure 1:**
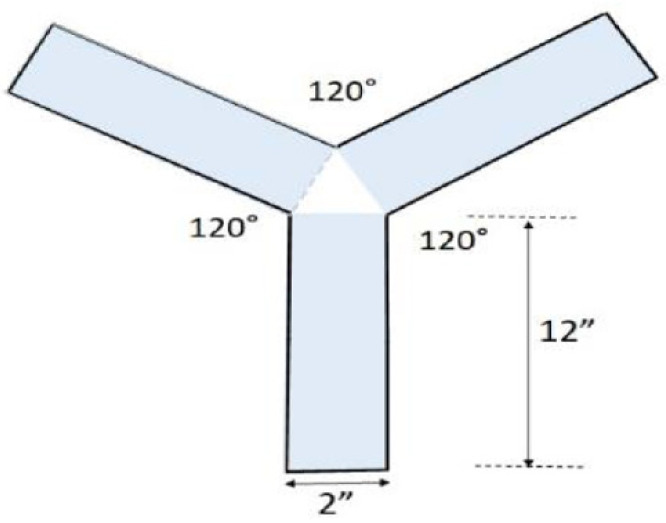
Y-maze.

A total of 128 1-week-old house crickets were used in this study. Crickets were housed in Y-mazes made with 12″ × 2″ × 2″ (L × H × W) inexpensive rectangular plastic tubes from Cleartec Packaging, Inc. (Park Hills, MO, USA) at angles of 120° relative to each other in a Y shape, leaving a triangular space (center zone) between the three tubes (Figure 1). Y-mazes were used because they are suitable for behavioral tests that do not induce significant stress to the crickets compared to other memory tests with apparatuses such as water mazes.

Small holes were drilled into the sides of the Y-mazes for aeration. Throughout the experiment, one house cricket was placed in the bottom arm of each Y-maze under a 6:18 light/dark schedule with a constant temperature of 75 °F. Crickets had constant access to food and water (in a gel form to protect crickets from drowning).

### 3.2. Nutrient Treatments

Multivitamins, zinc, polyphenols, omega-3 fatty acids, and probiotics were used in this study. All of these nutrients have been demonstrated to have a positive impact on memory and cognition when consumed independently.

#### 3.2.1. Multivitamins

Crickets were given one serving every two weeks, each serving including vitamin A (1200 μg), vitamin B1 (2.5 mg), vitamin B2 (2.5 mg), vitamin B3 (20 mg), vitamin B6 (3 mg), vitamin C (100 mg), vitamin D3 (10 μg), vitamin E (20 mg), and vitamin K1 (80 μg).

#### 3.2.2. Zinc

Crickets were given one serving every two weeks, each serving including zinc sulphate (ZnSO_4_) (220 mg).

#### 3.2.3. Polyphenols

Crickets were given one serving every two weeks, each serving (2.37 g) including turmeric extract (*Curcuma longa*), bitter orange, grape extract, organic decaf tea (leaf extract), olive extract, noni, pomegranate extract, hawthorn berry powder, apple extract, alfalfa, quercetin dihydrate, Aronia, acai (*Euterpe oleracea*), blueberry extract, and celery extract.

#### 3.2.4. Omega-3 Fatty Acids

Crickets were given one serving every two weeks, each serving including Omega-3 Phospholipid Peptide Complex (292 mg).

#### 3.2.5. Probiotics

Crickets were given one serving every two weeks, each serving including 50 billion colony forming units (CFUs) with 11 live bacterial strains: Lactobacillus rhamnosus, Lactobacillus acidophilus, Lactobacillus casei, Lactobacillus salivarius, Lactobacillus plantarum, Lactobacillus paracasei, Bifidobacterium longum, Bifidobacterium bifidum, Bifidobacterium lactis, Bifidobacterium breve, and Streptococcus thermophilus.

### 3.3. Groups

Based on the 31 possible combinations of multivitamins (Vit), zinc (Znc), polyphenols (Pol), omega-3 PUFAs (Puf), and probiotics (Pro), the 128 house crickets were divided into one control group and 31 experimental groups with four house crickets in each group (Table 1). Of the 128 house crickets, 124 (31 groups) were fed with various combinations of nutrients demonstrated to have a positive effect on cognitive performance, while the remaining four house crickets were fed with a normal diet (control group). Sufficient quantities of food and water were available for all house crickets.

### 3.4. Spatial Memory Testing

Spatial memory refers to the ability to memorize and recall locations and spaces, which is useful during navigation. With its simple, three-armed design, the Y-maze has traditionally been effective in evaluating spatial memory. In this experiment, Y-mazes were used to conduct weekly Alternation Tests and Recognition Memory Tests to assess spatial working memory.

#### 3.4.1. Habituation and Practice Sessions

During the one week of habituation, crickets were kept in the home arm of the Y-maze with the other arms blocked. This allowed crickets to become more comfortable in their environments. Within the home arm, crickets had access to water and normal food. At the end of the habituation period, the food and water were removed from the home arm and crickets were starved for one day and night in order to motivate them to collect the food from other arms of the Y-maze during the practice phase. After habituation was completed, crickets underwent practice sessions to ensure that they could collect food rewards from arms of the maze before testing and supplementation. During the week, crickets had two practice sessions with three trials, in which crickets collected a food reward from an open goal arm (no entry was provided to the alternate arm). The open goal arm was pseudo-randomly varied between trials in each practice session to prevent a preference for either one.

Immediately after completion of the one-week practice period, crickets were kept in the home arm of the Y-maze with the other arms blocked and fed according to their prescribed diet. Testing occurred twice a week, alternating between two tests that assess spatial memory using the Y-maze: the Alternation Test and the Recognition Memory Test. Testing continued throughout the feeding process to examine differences in the development of spatial memory over time with the nutrients provided, starting from a baseline established during the first week. The night before testing, crickets were slightly food-deprived so that they were motivated to explore the arms of the Y-maze.

#### 3.4.2. Alternation Test

**Figure 2:**
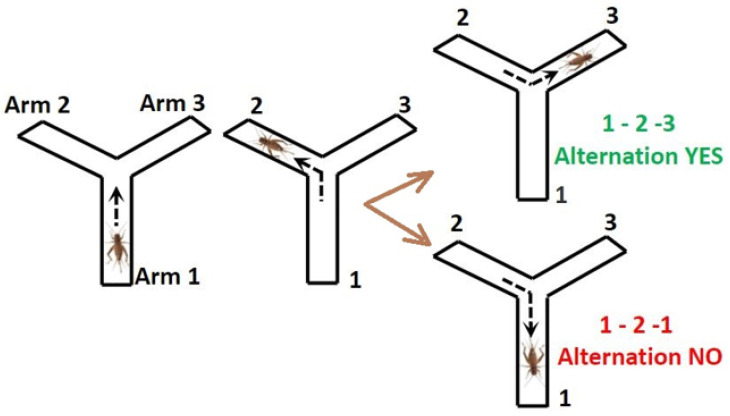
Alternation Test.

During the Alternation Test, crickets were allowed to explore all three arms of the Y-maze. Alternations were counted when the cricket explored a different arm each successive time in a set of three arm visits, such as arm 1 → arm 2 → arm 3. If the cricket explored an arm twice in the set of three arm visits, such as arm 1 → arm 2 → arm 1, this was not counted as an alternation (Figure 2). The Alternation Test is dependent on the fact that crickets habitually explore their least-recently visited location due to natural curiosity, which relies on their working memory of where they have previously visited. Each test consisted of six sets of three arm visits (18 arm visits total).

#### 3.4.3. Recognition Memory Test

**Figure 3:**
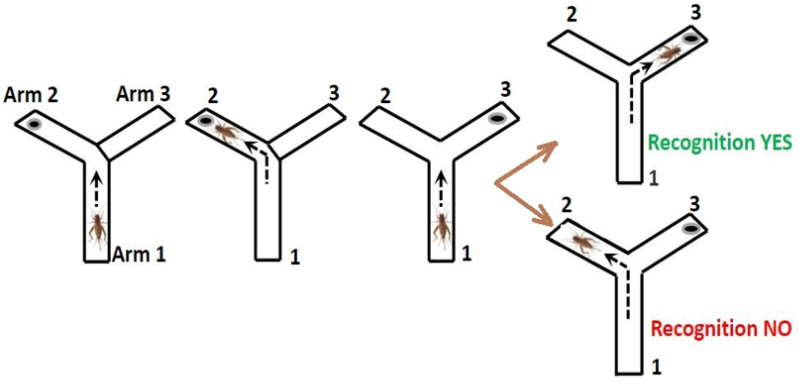
Recognition Memory Test.

During the Recognition Memory Test, crickets were given access to only one of arms 2 or 3 (for example, arm 2) of the Y-maze, where the food was placed (Figure 3). This arm was alternated among the six trials during each testing to avoid creating a bias for one arm. After obtaining the food, crickets were returned to arm 1 (the home arm), where they were sequestered for two minutes. During this time, food was placed in the unvisited arm (arm 3 in this example). After the two minutes were over, all arms were opened, and the crickets were able to freely explore the arm they have not visited, in which they would find the food. Thus, this test evaluated spatial memory by demonstrating whether the crickets had a recollection of which arm they had previously visited.

### 3.5. Potential Bias

**Figure 4:**
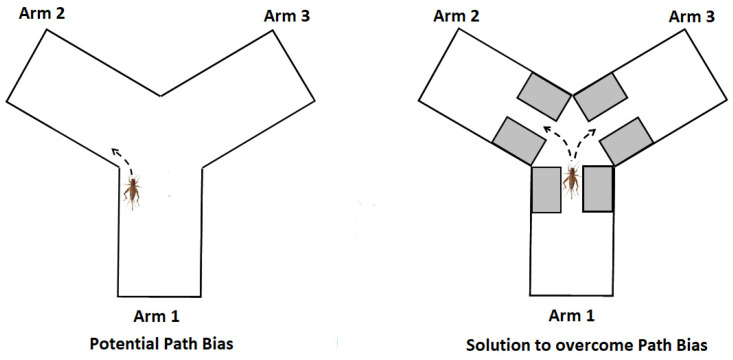
Path bias and solution to overcome path bias.

During the practice sessions, a possible “path bias” was observed affecting the crickets’ movement through the Y-maze. Crickets tended to travel along one edge of an arm, forming a path along that edge to one of the other two arms (Figure 4). For example, a cricket moving along the left side of the home arm would often follow that edge into the left arm (arm 2). An apparatus was constructed to overcome this “path bias”. Rectangular blocks of sponge (1.5″ × 2″ × 0.625″ L × H × W) were placed at either side of the arm just before the center zone, creating a narrow path (1.5″ × 2″ × 0.75″ L × H × W). Thus, even when the crickets followed one edge within their arm, they ultimately had to move to the center of the arm as they neared the center zone. This minimized path bias so that once each cricket reached the center zone, it had to explicitly choose which arm to explore.

### 3.6. Statistical Analysis

All data were analyzed using the Prism 8 data analysis program (GraphPad Software Inc., San Diego, CA, USA).

## 4. Results

**Figure 5:**
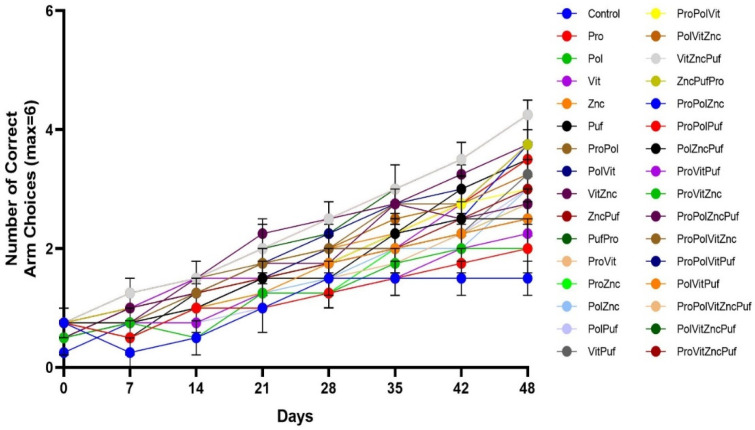
The effect of diet on performance in Recognition Memory Tests over time. Graphed is the mean performance of each group during each testing period with the standard error of the mean (SEM). All groups experienced improvement in performance over time.

**Figure 6:**
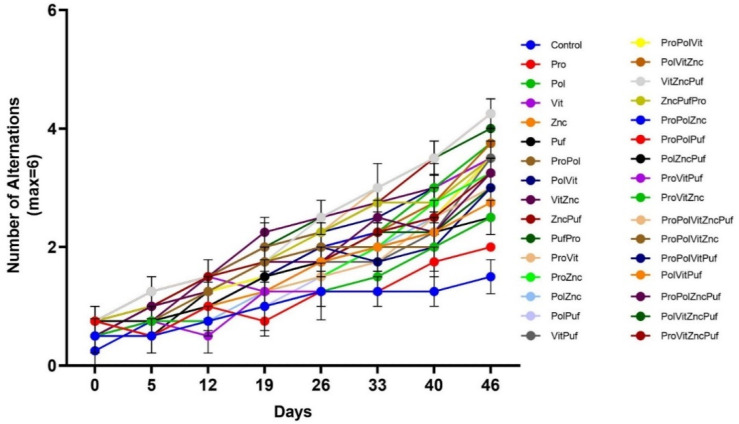
The effect of diet on performance in Alternation Tests over time. Graphed is the mean performance of each group during each testing period with the SEM. All groups experienced significant improvement in performance over time.

In both the Recognition Test (Figure 5) and the Alternation Test (Figure 6), all groups exhibited improvement in performance over time. A two-way repeated measures ANOVA showed that in the final results there was a significant main effect of diet on performance in both tests, as measured by the number of correct arm choices or alternations (*p* < 0.0001). Tukey tests were run along with the ANOVA to further examine differences among the means of individual combinations at the final trial. In the Alternation Test, the mean final performance of the Control varied significantly from the groups VitZncPuf, ProVitZncPuf, and ProPolVitZncPuf (*p* = 0.0194). There was no significant difference among these three groups. Similarly, in the Recognition Memory Test, the mean final performance of the Control varied significantly from the groups VitZncPuf, ProVitZncPuf, PolVitZncPuf, and ProPolVitZncPuf (*p* = 0.0194). There was no significant difference among these four groups.

**Figure 7:**
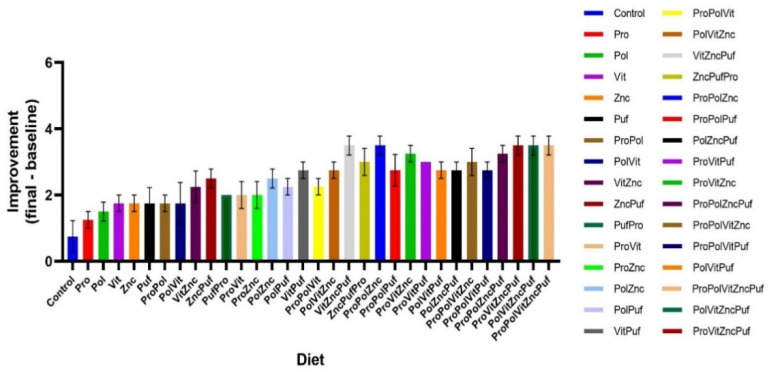
Improvement in performance in Recognition Memory Tests over time. Graphed is the mean improvement of each group with the SEM, calculated by final #-initial#.

**Figure 8:**
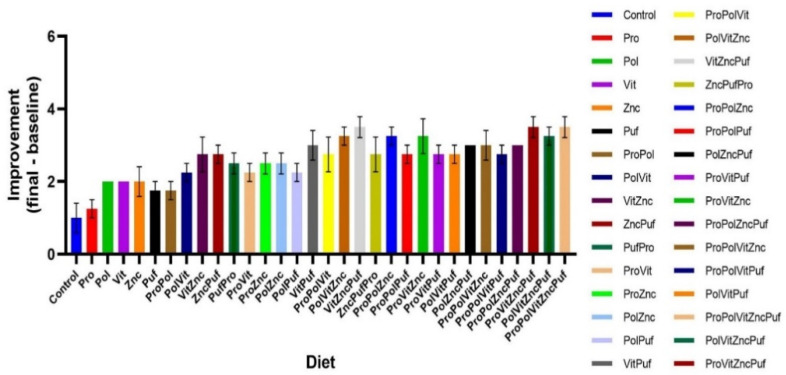
Improvement in performance in Alternation Tests over time. Graphed is the mean improvement of each group with the SEM, calculated by final #-initial#.

Further, the improvement of each group (final score-baseline score) was computed, and an ordinary one-way ANOVA was used to evaluate variance among these values for both the Recognition Test (Figure 7) and the Alternation Test (Figure 8). While the Control’s improvement differed significantly from many experimental groups in both tests, the most significant differences in the Alternation Test were between the Control and the groups VitZncPuf, ProVitZncPuf, and ProPolVitZncPuf (*p* < 0.0001). The most significant differences in the Recognition Memory Test were between the Control and the groups VitZncPuf, ProPolZnc, ProVitZncPuf, PolVitZncPuf, and ProPolVitZncPuf (*p* < 0.0001). In both tests, there was no significant difference among improvement scores within these winning groups.

**Figure 9:**
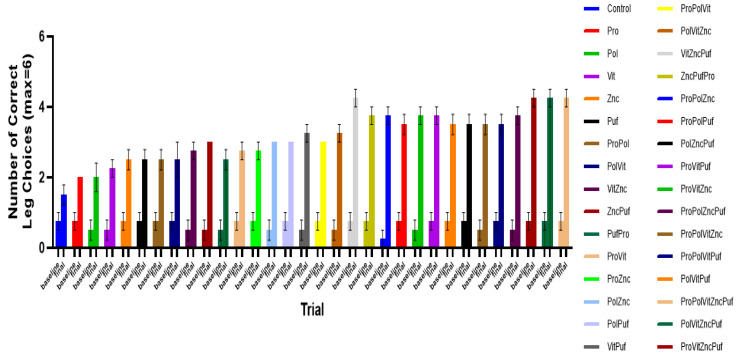
Baseline and final measures of performance in Recognition Memory Tests. Graphed are the mean number of correct arm/leg choices of each group at 0 days and 48 days since starting the specific diets. All groups exhibited improvements in performance.

**Figure 10:**
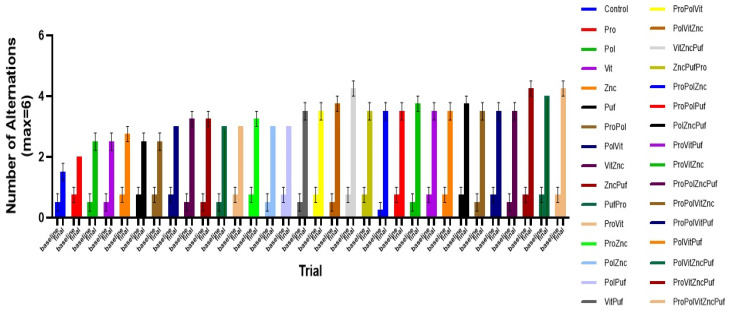
Baseline and final measures of performance in Alternation Tests. Graphed are the mean number of successful alternations of each group at 0 days and 48 days since starting the specific diets. All groups exhibited improvements in performance.

Figure 9 shows the mean number of correct arm choices of each group in the initial and final Recognition Memory Test since starting their specific diets. Figure 10 similarly shows the mean number of successful alternations of each group in the initial and final Alternation Test since starting their specific diets.

**Figure 11:**
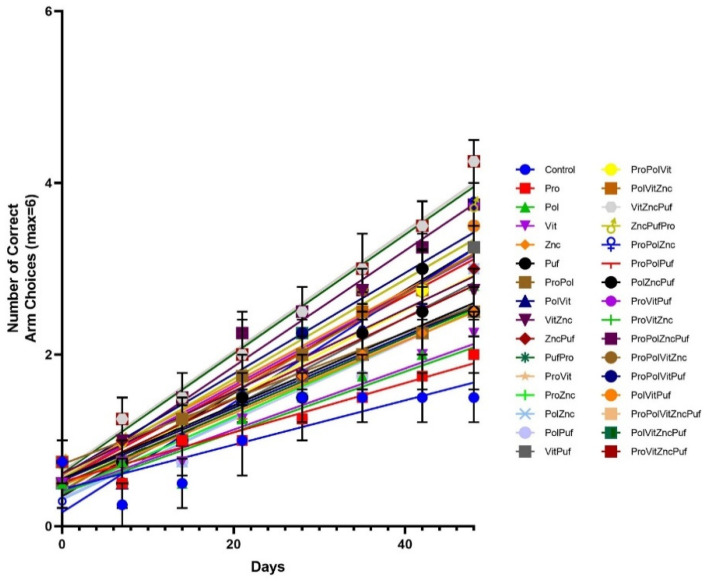
Recognition Memory Test: Regression. Graphed are the mean values for each group’s performance during each testing period with the SEM, as well as the line generated through regression analysis.

**Figure 12:**
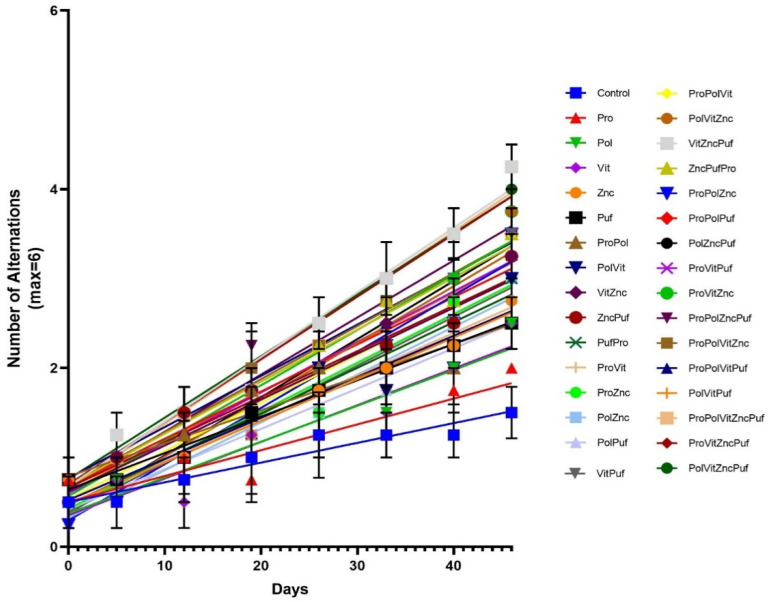
Alternation Test: Regression. Graphed are the mean values for each group’s performance during each testing period with the SEM, as well as the line generated through regression analysis.

Regression analysis was conducted to determine the relative magnitude of improvement over time, represented by the slope produced. Figure 11 and Figure 12 show the lines generated through regression analysis for each group in the Recognition Memory Test and the Alternation Test, respectively. In the Recognition Memory Test, the slopes were also significantly nonzero (*p* = 0.0005 for control group, *p* < 0.0001 for experimental groups) and significantly different from one another (*p* < 0.0001). In the Alternation Test, the slopes were significantly nonzero (*p* = 0.0025 for control group, *p* < 0.0001 for experimental groups) and significantly different from one another (*p* < 0.0001).

Table 2 shows the ranking of diets in order of their slope for both tests. Similar to the findings of the previous ANOVAs, the highest scoring diets shared by both tables were VitZncPuf (Alternation: slope = 0.07226, Recognition Memory: slope = 0.07001), ProPolVitZncPuf (Alternation: slope = 0.07182, Recognition Memory: slope = 0.07001), ProVitZncPuf (Alternation: slope = 0.06999, Recognition Memory: slope = 0.07001), and PolVitZncPuf (Alternation: slope = 0.06873, Recognition Memory: slope = 0.06956). The Control ranked the lowest in both tests (Alternation: slope = 0.02205, Recognition Memory: slope = 0.02590). Overall, regression analysis showed that the slopes were significantly nonzero and statistically different.

## 5. Discussion

This study tested the hypothesis that the combination of multivitamins, zinc, polyphenols, omega-3 fatty acids, and probiotics would best enhance spatial memory and cognitive performance. The results revealed that crickets consistently demonstrated more improvement in memory tests when fed nutrient-rich diets compared to crickets fed a normal diet, suggesting that these nutrients may indeed play a role in improving memory. While all of the nutrient combinations showed a benefit over the normal diet, the most significant improvement compared to the control was found in the VitZncPuf group (fed multivitamins, zinc, and omega-3 PUFAs), ProVitZncPuf group (fed probiotics, multivitamins, zinc, and omega-3 PUFAs), PolVitZncPuf group (fed polyphenols, multivitamins, zinc, and omega-3 PUFAs), and ProPolVitZncPuf group (fed probiotics, polyphenols, multivitamins, zinc, and omega-3 PUFAs). These four groups were also ranked highest in improvement based on linear regression analysis, with VitZncPuf group ranking only slightly higher than the ProVitZncPuf group, PolVitZncPuf group, and ProPolVitZncPuf group. However, as the post hoc Tukey test found no significant differences in the performance of subjects within these four groups (VitZncPuf, ProVitZncPuf, PolVitZncPuf, and ProPolVitZncPuf), the combination of multivitamins, zinc, and omega-3 PUFAs may be the most efficient option for improving memory, producing the greatest results with the least number of distinct nutrients. These results did not entirely support the experimental hypothesis, because although ProPolVitZncPuf was effective, its effects were not significantly higher than that of VitZncPuf, making multivitamins, zinc, and omega-3 PUFAs a potential winning combination.

Though previous studies did not focus on how combinations of nutrients interact to affect cognition, these results are consistent with findings regarding individual nutrients from this study’s winning combination (multivitamins, zinc, and omega-3 PUFAs). The current findings are supported by reports that prenatal and postnatal zinc supplementation in rats enhanced spatial learning, cognition, and locomotion [10]. Zinc deficiency has also been implicated in cognitive impairment, with improved learning and memory achieved following zinc supplementation [77,92]. Zinc is known to be concentrated in the CNS, particularly in the hippocampus, where it is involved in synaptic transmission [10]. These findings are further supported by another study of rats that developed zinc deficiencies following 145 days of a low-zinc diet, after which they received zinc-enriched yeast for 55 days. Although the zinc-deficient rats had impaired learning and memory, these effects were mitigated through administration of zinc-enriched yeast [93]. However, it remains unknown whether these findings can be observed in humans as well, as a study of 602 children found that administration of 30 mg of zinc for six months had little effect on improving cognition [94].

This study’s results are also consistent with findings that vitamin intake can benefit cognition in people without dementia [49]. A previous study also found significantly improved cognition in aged mice that received vitamin E and C for 60 days [95]. Another study observed cognitive improvements in 48 adults aged 65 who received multivitamins (vitamin B6, B12, and folic acid) for 12 weeks [96]. A study of 114 people with hyperhomocysteinemia also found that supplements of vitamin B6, B12, and folic acid improved cognitive function [97]. Another study using 32 house crickets examined the effects of combinations of polyphenols, probiotics, and multivitamins on spatial memory and cognitive performance and found that the combination of probiotics and multivitamins led to the most improvement [98]. Present results are also consistent with evidence that omega-3 fatty acids improve spatial memory [99,100,101]. Moreover, omega-3 fatty acids are known to regulate expression of several genes associated with apoptosis and oxidative stress [102]. Low dietary omega-3 PUFAs and low plasma DHA have also been found to lead to behavioral deficits and low omega-3 PUFA levels in the brain [103].

The subjects in this study were limited to house crickets as crickets have been established as the best insect model to investigate learning and memory [104]. While data on the time that crickets took to travel through the Y-maze may be useful in analyzing memory, this study did not include these measurements, due to the crickets’ inconsistent pauses during their exploration of the arms of the Y-maze. There were no outstanding risks to the subjects, as the crickets were adequately fed with necessary nutrients. No physical stress was applied to the subjects throughout the study. Quencher was used as the water source to maintain cleanliness and protect the crickets from drowning in water when they were young. The National Institutes of Health (NIH) Guide for the Care and Use of Laboratory Animals (8th Edition) was followed during testing.

Future investigations may replicate this experiment with mice using a Morris water maze. Other nutrients, such as iron or fiber, may also be tested, which could reveal an even more effective combination of dietary supplements to improve cognitive performance. Further investigations may also examine combinations of individual vitamins within the multivitamin cocktail used in this study. In addition, future research may directly study the effect of nutrition on human cognitive performance, particularly during childhood learning. This may be examined through natural studies measuring nutrition and performance and evaluating correlations between these factors and income level. Such studies could illustrate the effect of socioeconomic disparities on nutrition and learning, ultimately pointing to changes that can be made in mass nutrition.

## 6. Implications

These findings suggest potential ways of efficiently using nutrition in dietary strategies to support learning and cognition in humans. This research has implications for food insecurity, as many people lack access to nutritious foods that support learning and cognitive performance. This may be crucial in the context of childhood education, during which barriers to food access could affect learning and thus affect academic outcomes. This study may direct further research developing more detailed nutritional plans for supporting cognition, as well as informing larger-scope changes to be made in food access and mass nutrition to better support childhood learning.

## 7. Conclusions

Maintaining a nutritious diet necessary for health can be challenging for those facing poverty or food insecurity due to limited resources, stress, and competing priorities. Knowing how food can affect cognition informs strategies to protect and improve neuronal function through modifying diet and mass nutrition. This study investigated the best combination of nutrients for enhanced cognitive performance and memory in the house cricket. All of the nutrient combinations tested demonstrated a benefit over the control diet, but the most significant improvement compared to the control was found in the combination of multivitamins, zinc, and omega-3 fatty acids (VitZncPuf), the combination of probiotics, multivitamins, zinc, and omega-3 PUFAs (ProVitZncPuf), the combination of polyphenols, multivitamins, zinc, and omega-3 PUFAs (PolVitZncPuf), and the combination of probiotics, polyphenols, multivitamins, zinc, and omega-3 PUFAs (ProPolVitZncPuf). Since this study found no significant difference between the performance and improvement of subjects within these four groups, the combination of multivitamins, zinc, and omega-3 fatty acids (VitZncPuf) was concluded to be the most effective option for improving memory and cognitive performance. While the group fed multivitamins, zinc, polyphenols, omega-3 fatty acids, and probiotics produced improvement, its improvement was not significantly higher than that of the group fed multivitamins, zinc, and omega-3 fatty acids. To strengthen or corroborate the findings of this study, future investigations may replicate it with mice using either a Y-Maze or a Morris water maze apparatus.

## Figures and Tables

**Table 1:** Groups.

Group	Group Label	Diet
Group 1	Control	Normal diet
Group 2	Pro	Probiotics
Group 3	Pol	Polyphenols
Group 4	Vit	Multivitamins
Group 5	Znc	Zinc
Group 6	Puf	Omega-3 PUFAs
Group 7	ProPol	Probiotics and Polyphenols
Group 8	PolVit	Polyphenols and Multivitamins
Group 9	VitZnc	Multivitamins and Zinc
Group 10	ZncPuf	Zinc and Omega-3 PUFAs
Group 11	PufPro	Omega-3 PUFAs and Probiotics
Group 12	ProVit	Probiotics and Multivitamins
Group 13	ProZnc	Probiotics and Zinc
Group 14	PolZnc	Polyphenols and Zinc
Group 15	PolPuf	Polyphenols and Omega-3 PUFAs
Group 16	VitPuf	Multivitamins and Omega-3 PUFAs
Group 17	ProPolVit	Probiotics, Polyphenols, and Multivitamins
Group 18	PolVitZnc	Polyphenols, Multivitamins, and Zinc
Group 19	VitZncPuf	Multivitamins, Zinc, and Omega-3 PUFAs
Group 20	ZncPufPro	Zinc, Omega-3 PUFAs, Probiotics
Group 21	ProPolZnc	Probiotics, Polyphenols, and Zinc
Group 22	ProPolPuf	Probiotics, Polyphenols, and Omega-3 PUFAs
Group 23	ProVitZnc	Probiotics, Multivitamins, and Zinc
Group 24	ProVitPuf	Probiotics, Multivitamins, and Omega-3 PUFAs
Group 25	PolVitPuf	Polyphenols, Multivitamins, and Omega-3 PUFAs
Group 26	PolZncPuf	Polyphenols, Zinc, and Omega-3 PUFAs
Group 27	ProPolVitZnc	Probiotics, Polyphenols, Multivitamins, and Zinc
Group 28	ProPolVitPuf	Probiotics, Polyphenols, Multivitamins, and Omega-3 PUFAs
Group 29	ProPolZncPuf	Probiotics, Polyphenols, Zinc, and Omega-3 PUFAs
Group 30	ProVitZncPuf	Probiotics, Multivitamins, Zinc, and Omega-3 PUFAs
Group 31	PolVitZncPuf	Polyphenols, Multivitamins, Zinc, and Omega-3 PUFAs
Group 32	ProPolVitZncPuf	Probiotics, Polyphenols, Multivitamins, Zinc, and Omega-3 PUFAs

**Table 2:** The ranking of diets in order of their slope for the (**a**) Alternation Test and (**b**) Recognition Memory Test.

(a)		(b)	
Ranking	Combination	Ranking	Combination
1	VitZncPuf	1	VitZncPuf ProVitZncPuf ProPolVitZncPuf
2	ProPolVitZncPuf	2	PolVitZncPuf
3	ProVitZncPuf	3	ProPolZncPuf
4	PolVitZncPuf	4	ProPolZnc
5	PolZncPuf	5	PolZncPuf
6	ProPolZncPuf	6	ProPolVitPuf
7	PolVitZnc	7	ProPolVitZnc
8	ProPolZnc	8	ZncPufPro
9	ProVitZnc	9	PolVitZnc
10	ProPolVitZnc	10	ProVitZnc
11	ZncPufPro	11	ProVitPuf
12	ProPolVitPuf	12	PolVitPuf
13	ProVitPuf	13	ProPolPuf
14	PolVitPuf	14	VitPuf
15	VitPuf	15	ProPolVit
16	ProZnc	16	VitZnc
17	ProPolVit	17	PolZnc
18	PolZnc	18	ZncPuf
19	ProPolPuf	19	PolPuf
20	VitZnc	20	ProZnc
21	PufPro	21	Puf
22	ZncPuf	22	PufPro
23	ProVit	23	PolVit
24	Znc	24	Znc
25	PolVit	25	ProVit
26	PolPuf	26	ProPol
27	Vit	27	Vit
28	Puf	28	Pol
29	Pol	29	Pro
30	ProPol	30	Control
31	Pro		
32	Control		
